# Nasopharyngeal cancer in monozygotic twins: A case report and review of the role of genetics and the environment

**DOI:** 10.1016/j.ijscr.2021.106371

**Published:** 2021-09-06

**Authors:** Ayshah Almahboob, Ahmad Alroqi, Mohammed I. Almohizea

**Affiliations:** Department of Otolaryngology, College of Medicine, King Saud University, Riyadh, Saudi Arabia

**Keywords:** Twins, NPC, Monozygotic, Genetics, Environment, Screening

## Abstract

**Introduction and importance:**

Nasopharyngeal carcinoma (NPC) is considered a rare malignant head and neck tumour. However, the importance of genetics and environmental factors in the epidemiology of NPC is still unclear. Twins represent an excellent study population for genetic epidemiology; this is especially true of monozygotic-type twins because they are genetically identical. The difference in cancer occurrence between monozygotic twins is typically interpreted as a result of possible environmental factors.

**Case presentation and clinical discussion:**

We present the first case report of monozygotic twins with NPC. The twins' significant features are homogenous presentation, tumour location (both left-sided) and identical histology; therefore, the prognoses may be similar. Environmental factors could not be addressed in these twins because they shared the same background, and at the same time, they had no potential known contributing factors.

**Conclusion:**

Having one of the twins affected is a strong and easily recognisable risk factor for developing NPC in the other. This strong association suggests the need for regular screening of the second twin for early diagnosis of NPC.

## Highlights

• The importance of genetics and environmental factors in the epidemiology of NPC is still unclear.
• Having one of the twins affected is a strong and easily recognisable risk factor for developing Nasopharyngeal carcinoma in the other.
• This strong association suggests the need for regular screening of the second twin for early diagnosis of Nasopharyngeal carcinoma.

## 1 Introduction

Nasopharyngeal carcinoma is considered a rare malignant head and neck tumour; however, it is common in specific geographical distributions, such as Southeast Asia [1]. The importance of genetic factors in NPC epidemiology has been studied extensively, and familial clustering of NPC has been reported in the literature among Chinese and low-risk populations [2]. However, NPC occurrence in twins is extremely rare.

Twins demonstrate an excellent study population for genetic epidemiology; this is particularly true of monozygotic-type twins because they are genetically identical, whereas dizygotic twins share only half of their genes [3]. A difference in cancer occurrence between monozygotic twins is typically interpreted as a possible environmental factor [4]. In addition to hereditary factors, long-term cigarette smoking, consumption of salted fish and exposure to wood dust have also been associated with the NPC incidence [5].

To our knowledge, NPC in monozygotic twins has not been reported previously in the literature. This report introduces the first case of NPC in monozygotic twins, with an analysis of their background history.

This case report has been written in line with the 2020 SCARE Criteria [6].

## 2 Case report

### 2.1 Case 1

A 34-year-old male presented in February 2019 with a complaint of spitting up blood through the mouth, occasional left self-limited epistaxis for 4 months and a 6-month history of left hearing loss. He was otherwise healthy, with his surgical history remarkable only for right tympanoplasty and septoplasty in 2011. Nasal endoscopy revealed a left-sided fungating mass; it was irregular on the surface, centred on the left nasopharynx and obstructing the eustachian tube (ET). The right side of the nasopharynx was spared. The patient had left otitis media with effusion (OME) with tympanometry of type C. The rest of the examination, including that of the neck and cranial nerves, was unremarkable.

Computerised tomography (CT) of the head and neck with contrast demonstrated a heterogeneous enhancing mass occupying the posterior wall of the nasopharynx, mainly towards the left side, obstructing the ET and ipsilateral fossa of Rosenmüller. Adjacent bony structures were not involved, but there was bilateral enhancement of the lymph nodes, with the largest on the left side at level II, measuring around 1 × 0.8 cm. A biopsy was obtained from the nasopharynx, and a diagnosis of nasopharyngeal cancer was made (non-keratinising, undifferentiated squamous cell carcinoma, T1/N2/M0, AJCC stage III). The patient underwent radio-chemotherapy, and the post-treatment positron emission tomography scan was unremarkable. Since then, the patient has been followed up regularly, and he has remained free of disease for 2 years.

### 2.2 Case 2

A 34-year-old male, the twin of case 1, presented in February 2021 with a complaint of painless progressive left neck mass and left hearing loss for 2 months with a history of spitting blood orally. Nasal endoscopy revealed a fungating mass with an irregular surface centred in the left nasopharynx and obstructing ET. The right side of the nasopharynx was spared. There was OME in the left ear (tympanometry of type B with normal external canal volume), and the neck showed a level II ipsilateral lymph node that was firm, not tender, and not mobile, with an ill-defined border.

Contrast CT of the head and neck showed a left-sided posterior nasopharyngeal mass obstructing the left fossa of Rosenmüller and sparing the right tubal orifice. Enhanced ipsilateral lymph nodes were observed at levels II and III, with the largest measuring 2.4 × 3.8 cm. A biopsy was taken from the nasopharynx and confirmed identical histopathology as in case 1 (non-keratinising undifferentiated squamous cell carcinoma), with positive malignant cells found in the fine-needle aspiration of the suspected lymph nodes (T1/N1/M0), stage II.

### 2.3 Social history and environmental risk factors

The cases are monozygotic male twins living in the same country in a non-industrial area. Both are non-smokers, and they have no history of radiation.

### 2.4 Family history

There was no family history of head and neck malignancy.

## 3 Discussion

NPC is a rare tumour throughout the world [1]; the relative contribution of genetic and environmental factors in the causation of NPC have been hypothesised, but they are still unproven [7]_._ We introduce this first case report of monozygotic twins with NPC. The impressive features in these twins were the homogenous presentation, tumour location (both left sided) and identical histology; however, the development of symptoms was not simultaneous in the two cases.

The strong support for the significance of environmental factors was due to an observation in the United States (U.S.) of a lower incidence of NPC among Chinese men born in that country, in contrast to Chinese immigrants with similar demographic characteristics [7]_._ Various environmental factors have been suggested as possibly linked to an increased risk of NPC. For instance, an increased incidence of this cancer was observed among Chinese people who had consumed salted fish during their early years [8]. In addition, being a smoker or having excessive exposure to smoke was associated with a high risk for NPC among the Chinese population [8]_._ In the present report, it is hard to assess the role of environmental factors in the pathogenesis of NPC because the affected twins shared the same background; at the same time, they did not carry any known potential factors for the development of this cancer.

NPC is extremely rare in most countries of the world, so there have been fewer investigations of familial aggregation compared with other cancers [9]. However, the importance of genetic factors has been proposed in multiple studies that demonstrated a higher risk of NPC with specific human leukocyte antigen (HLA) histocompatibility antigens, such as 6p22 and 3p21 [10], [11]. In contrast, some studies have suggested that this association is more linked to Epstein–Barr virus (EBV) because HLA alleles are crucial for proper presentation of viral antigens to the immune system. Currently, there are no conclusive results related to this issue [12].

The histology of familial NPC has been reported to involve the undifferentiated (non-keratinising) type more frequently [2], and this was observed in our case. This can be compared with the sporadic type (differentiated type, World Health Organization [WHO] type 1); this is seen in Caucasian people, and it is more frequent in general.

Our findings showed that, if one of the twins had NPC, the other would be at a greater risk of developing this malignancy. Technological advances in clinical examination modalities, such as the use of nasal endoscopy and radiological imaging, have improved treatment outcomes in the modern era [13]. Most authors advocate for screening after the age of 30 years, representing the age at which familial NPC is most likely to present. Few authors have suggested screening below this age because of the scarcity of cases reported to have cancer below the age of 30 years [14]. Enhanced awareness of NPC symptoms by family members and seeking medical attention in a timely manner will facilitate earlier diagnosis [14].

To our knowledge, only a few studies have published reports of twins with NPC; in some of those studies, the affected dizygotic twins presented simultaneously or 1 month apart [2], [3]. In contrast, in our case, the second twin presented 2 years after the first twin's diagnosis, and this highlights the need for long-term screening duration because the cancers might not exhibit concurrent clinical manifestations. In addition, we observed that NPC in monozygotic twins may have a higher possibility of identical histology and presentation, and therefore, an identical prognosis, compared with previously reported pathological diagnoses of dizygotic twins [2], [7], [14]. Fortunately, the second twin in this report had an earlier stage of NPC, probably due to enhanced awareness of this cancer in his family member.

In the future, familial case-based chromosome analysis studies are required to locate the genetic susceptibility to NPC. This will eventually guide the understanding of the genetic mechanism responsible for the occurrence of NPC.

## 4 Conclusion

A pair of monozygotic twins is an ideal study population for genetics epidemiology in cancer occurrence; having one of the twins affected is a strong and easily recognisable risk factor for developing NPC. This strong association suggests the need for regular screening of the second twin for early diagnosis of this cancer. Educating the population about the tendency of NPC to occur in the second twin would improve secondary prevention.

## Sources of funding

N/A

## Ethical approval

N/A

## Consent

Written informed consent was obtained from the patient for publication of this case report. A copy of the written consent is available for review by the Editor-in-Chief of this journal on request.

## Research registration

N/A

## Guarantor

DR.Ayshah Almahboob, MBBS, Department of Otolaryngology, College of medicine, King Saud University, Riyadh, Saudi Arabia.

## Provenance and peer review

Not commissioned, externally peer-reviewed.

## CRediT authorship contribution statement

1.Ayshah Almahboob: *.

First author, Data curation, Writing- Reviewing and Editing manuscript.

2.Ahmad Alroqi: *.

Conceptualization, Data curation, Reviewing and Editing manuscript.

3.Mohammed Almohizea:

Supervision.

*Both authors contribute equally in writing this manuscript and considered a joint-authorship.

## Declaration of competing interest

N/A
